# *In vitro* cytocidal effects of the essential oil from *Croton cajucara* (red sacaca) and its major constituent 7- hydroxycalamenene against *Leishmania chagasi*

**DOI:** 10.1186/1472-6882-13-249

**Published:** 2013-10-02

**Authors:** Igor A Rodrigues, Mariana M B Azevedo, Francisco C M Chaves, Humberto R Bizzo, Suzana Corte-Real, Daniela S Alviano, Celuta S Alviano, Maria S S Rosa, Alane B Vermelho

**Affiliations:** 1 Departamento de Microbiologia Geral, Instituto de Microbiologia Paulo de Góes/UFRJ, 21941-590 Rio de Janeiro, RJ, Brazil; 2 Programa de Pós Graduação PAPD CAPES/FAPERJ, Instituto de Microbiologia Paulo de Góes/UFRJ, 21941-590 Rio de Janeiro, RJ, Brazil; 3 Programa de Pós Graduação Ciências dos Alimentos do Instituto de Química/UFRJ, 21941-590 Rio de Janeiro, RJ, Brazil; 4 Embrapa Amazônia Ocidental, CP 319, Manaus, AM 69010-970, Brazil; 5 Embrapa Agroindústria de Alimentos, 23020-470, Rio de Janeiro, RJ, Brazil; 6 Laboratório de Biologia Estrutural, Plataforma Instituto Oswaldo Cruz (IOC), Manguinhos, 21040-360 Rio de Janeiro, RJ, Brazil

## Abstract

**Background:**

Visceral leishmaniasis is the most serious form of leishmaniasis and can be lethal if left untreated. Currently available treatments for these parasitic diseases are frequently associated to severe side effects. The leaves of *Croton cajucara* are used as an infusion in popular medicine to combat several diseases. Previous studies have demonstrated that the linalool-rich essential oil from *C. cajucara* (white sacaca) is extremely efficient against the tegumentary specie *Leishmania amazonensis*. In this study, we investigated the effects of the 7-hydroxycalamenene-rich essential oil from the leaves of *C. cajucara* (red sacaca) against *Leishmania chagasi,* as well as on the interaction of these parasites with host cells.

**Methods:**

Promastigotes were treated with different concentrations of the essential oil for determination of its minimum inhibitory concentration (MIC). In addition, the effects of the essential oil on parasite ultrastructure were analyzed by transmission electron microscopy. To evaluate its efficacy against infected cells, mouse peritoneal macrophages infected with *L. chagasi* promastigotes were treated with the inhibitory and sub-inhibitory concentrations of the essential oil.

**Results:**

The minimum inhibitory concentrations of the essential oil and its purified component 7-hydroxycalamenene against *L. chagasi* were 250 and 15.6 μg/mL, respectively. Transmission electron microscopy analysis revealed important nuclear and kinetoplastic alterations in *L. chagasi* promastigotes. Pre-treatment of macrophages and parasites with the essential oil reduced parasite/macrophage interaction by 52.8%, while it increased the production of nitric oxide by *L. chagasi*-infected macrophages by 80%.

**Conclusion:**

These results indicate that the 7-hydroxycalamenene-rich essential oil from *C. cajucara* is a promising source of leishmanicidal compounds.

## Background

Human visceral leishmaniasis (HVL) or kala-azar is an often lethal infectious disease. About 500,000 new cases of visceral leishmaniasis are reported worldwide each year [1]. In Brazil, approximately 4,000 people are infected with leishmaniasis each year, and 10.5% die from the disease. The disease is more common in the Northeastern part of the country, but it extends to tropical forest regions and to some major industrial cities in the Southeastern region [2,3].

Conventional chemotherapy, one of the most common treatments for leishmaniasis, is highly toxic and fails in approximately 10% of cases [2]. Among the chemotherapeutic agents used to treat the disease, the pentavalent antimonials are still the first choice. However, the current scenario of drug development for leishmaniasis is more promising than a few decades ago. Recently, potential therapies for visceral leishmaniasis have been introduced, including liposomal amphotericin B, paromomycin, and miltefosine [4]. Despite the advances, both the conventional treatments and the new chemotherapeutic agents have a number of important disadvantages such as severe side effects and high cost.

**Figure 1:**
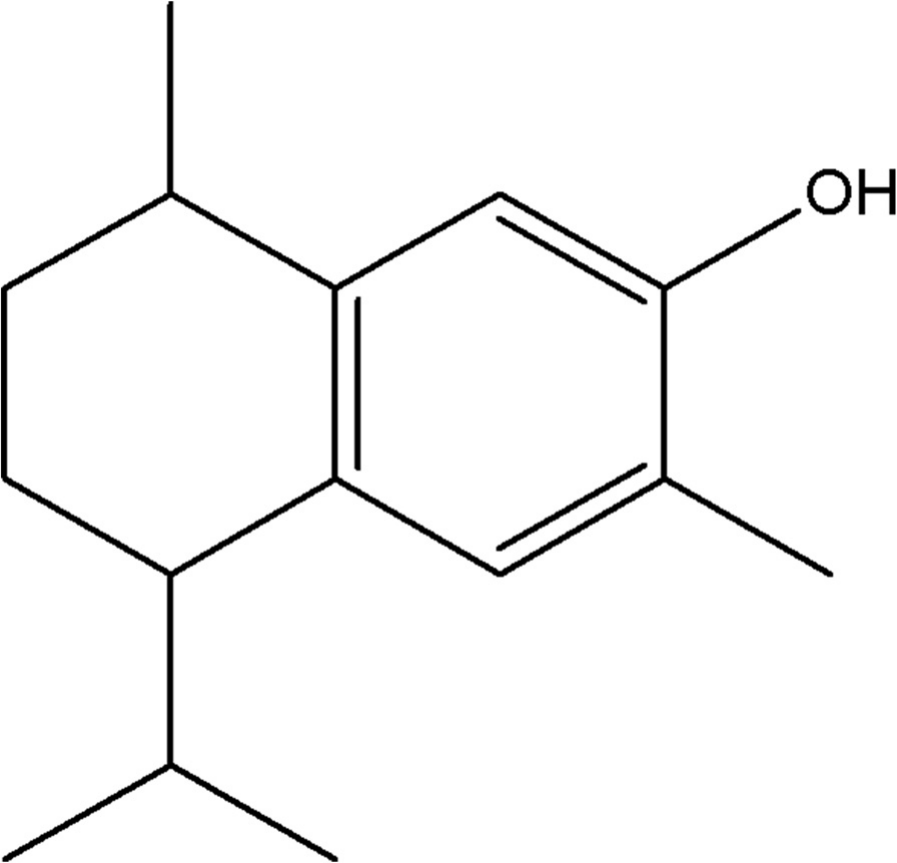
Chemical structure of 7-hydroxycalamenene.

Given the limitations of treatment against visceral leishmaniasis, there is a need for development of new drugs. The use of antimicrobials and other drugs derived from plants has been considered promising. *Croton cajucara* Benth. (family Euphorbiaceae), locally known as “sacaca”, is a plant found in the Amazon region that has been used in folk medicine against gastrointestinal and liver disorders, diabetes, and for cholesterol reduction. Two morphotypes were identified, namely white sacaca and red sacaca [5,6]. The essential oils of white sacaca and red sacaca were classified in two groups: one rich (up to 45%) in linalool [7], and other containing up to 44% of an aromatic sesquiterpene, isolated and identified by NMR as 7-hydroxycalamenene (Figure 1) [8].

Prompted by the fact that the essential oils extracted from leaves of white and red sacaca present antimicrobial properties, being effective against several microorganisms, including *L. amazonensis*[9], *Staphylococcus aureus* MRSA, *Mycobacterium smegmatis*, *M. tuberculosis*, *Rhizopus oryzae*, and *Candida albicans*[5-7], we decided to investigate the effects of the 7-hydroxycalamenene-rich essential oil of red sacaca against *Leishmania chagasi* parasites. In addition, the effects of the essential oil on the interaction of these parasites with mammalian host cells were evaluated.

## Methods

### Chemicals

Culture media were purchased from Difco (Sparks, MD 21152, USA). Reagents used in electrophoresis and molecular mass standards were acquired from Amersham Life Science (Little Chalfont, England). All other reagents were analytical grade.

### Plant material, essential oil extraction and 7-hydroxycalamenene purification

All samples were kept in a germplasm bank under the same cultivation practices. Leaves of *C. cajucara* were collected between 08:00 and 09:00 AM. Voucher specimens were deposited at the Embrapa Occidental Amazon Herbarium (registry IAN 165013). The oils were obtained by hydrodistillation in a modified Clevenger apparatus for 4 hours, carefully separated, and stored in opaque glass vials in a refrigerator (−10°C) prior to analysis and biological assays [7].

The isolation of 7-hydroxycalamenene was performed by preparative column chromatography on silica gel (Merck, 70–230 mesh), eluting with hexane and hexane-ethyl acetate mixtures.

### Analysis of the essential oil by GC-MS

The essential oils were analyzed at GC-MS under the following conditions: the oven temperature was programmed from 60°C to 240°C at 3°C/min, and helium was the carrier gas (at 1.0 mL/min). One microliter of 1% solution of the oil in dichloromethane was injected in split mode (1:100). Mass spectra were obtained in an Agilent 5973N system, fitted with a low bleeding 5% phenyl/95% methylsilicone (HP-5 MS, 30 m × 0.25 mm × 0.25 μm) fused silica capillary column, operating in electronic ionization mode (EI) at 70 eV, with scan mass range of 40–500 m/z. Sampling rate was 3.15 scan/s. Ion source was kept at 230°C, mass analyzer at 150°C, and transfer line at 260°C. Linear retention indices (LRI) were measured by injection of a series of *n*-alkanes (C_7_-C_30_) in the same column and conditions as described above and compared with reference data [8].

### Analyses of 7-hydroxycalamenene by GC

In order to evaluate the degree of purity of the isolated material, 7-hydroxycalamenene was analyzed on an Agilent (Palo Alto, CA, USA) 6890N gas chromatograph fitted with a 5% phenyl—95% methylsilicone (HP-5, 30 m × 0.32 mm × 0.25 μm) fused silica capillary column. The oven temperature was programmed from 60°C to 240°C (3°C/min), and hydrogen was used as carrier gas (1.4 mL/min). It was injected 1.0 μL of a 1% solution of the 7-hydroxycalamenene in dichloromethane, in split mode (1:100). Injector was kept at 250°C and detector (FID) at 280°C. All analyses were performed in triplicate.

The percentage was calculated as % peak area of GC–FID. The material purity was over 80%.

### Parasite culture

Promastigote forms of *Leishmania (L.) chagasi* MHOM/BR/1974/PP75 were obtained from *Leishmania* Type Culture Collection (LTCC) of Oswaldo Cruz Institute/Fiocruz (Rio de Janeiro, RJ, Brazil). Parasites were maintained by weekly transfers in PBHIL medium supplemented with 10% fetal bovine serum (FBS), at 28°C [10]. Parasites were maintained infective by periodical macrophage infection.

### Antileishmanial activity

To evaluate the minimum growth-inhibitory concentration (MIC), promastigote forms of *L. chagasi* (10^6^ parasites/mL) were incubated in fresh medium in the presence of several concentrations (1–1000 μg/mL) of essential oil and its 7-hydroxycalamenene-rich purified fraction (80%). After 120 h incubation, parasite viability was determined using the microplate method based on the reduction of resazurin as described [11]. Alternatively, cells were centrifuged and washed three times in PBS (150 mM NaCl; 20 mM phosphate buffer, pH 7.2) and resuspended in fresh culture medium without the plant extract, to evaluate the leishmanicidal or leishmanistatic effect. The 50% lethal dose (LD_50_) was determined by logarithmic regression analysis of the data obtained as described above.

### Transmission electron microscopy

Parasites at early stationary phase of growth were harvested, washed twice with PBS, and incubated or not with 250 μg/mL (MIC concentration) of the essential oil at 28°C for 24 hours. Cells were washed twice in PBS and then fixed with 2.5% glutaraldehyde in 0.1 M sodium cacodylate buffer containing 3.5% sucrose, pH 7.4, at 4°C for 60 min. Parasites were then rinsed in PBS pH 7.4 and pelleted by centrifugation. The pellets were then post-fixed with a 1% osmium tetroxide and potassium ferrocyanide solution for 1 hour, dehydrated sequentially in acetone, and then embedded in Epon 812. Ultrathin sections were cut using an LKB ultramicrotome and collected on copper grids. Sections were stained with uranyl acetate and lead citrate and were observed in a Jeol JEM1011 transmission electron microscope.

### Peptidase activity of *Leishmania chagasi* by gelatin–SDS-PAGE

Promastigote forms of *L. chagasi* (2.0x10^6^ parasites/mL) were harvested at the log phase of growth, washed two times in PBS by centrifugation, and then incubated in PBS pH 6.8 with the essential oil (250 μg/mL) at 28°C for 1 hour. Controls were prepared without the essential oil. Cells were centrifuged and the supernatants collected and concentrated by dialysis (cut-off 9000 Da) against polyethylene glycol 4000 for 12 hours at 4°C. Cells were disrupted at 4°C by four 15-s periods of ultrasound treatment with 1-min intervals. The extracts were centrifuged and supernatant aliquots were stored at −60°C. Polyacrylamide gels containing 0.2% copolymerized gelatin as substrate were loaded with 28 μg of proteins (cellular extract and supernatants) per slot. After electrophoresis at a constant voltage of 200 V at 4°C, gels were soaked in 2.5% Triton X-100 (1 h) to remove SDS, and then incubated for 18 h at 37°C in 0.1 M phosphate buffer, pH 5.5, in the absence or in the presence of protease inhibitors (1 mM E-64 and 1 mM phenanthroline). Gels were stained for 1 h with 0.2% Comassie brilliant blue R-250 in methanolacetic acid-water (50:10:40) and de-stained to expose proteolytic bands in the same solvent. The relative molecular mass of the peptidases was calculated by comparison with the mobility of low molecular mass standards. Peptidase activity was estimated by the intensity of the bands using ImageJ program (NIH).

### Peritoneal mouse macrophages and cytotoxity assay

Female Swiss mice (6–8 weeks old) from a colony at the General Microbiology Department/UFRJ animal house facility were used in all of the experiments. The animals were maintained at 21 ± 2°C, on a 12hs light/dark cycle, with food and water until 1 h prior to the experimental procedures. The animals were killed according to the federal guidelines and institutional policies by cervical dislocation. The procedures were approved by the Fiocruz Commitee of Ethics for the Use of Animals (resolution 242/99, license LW 2/12). Peritoneal mouse macrophages were collected in cold PBS and allowed to adhere onto coverslips placed in 24-well culture plates, for 30 min at 37°C and 4% CO_2_. For the cytotoxity assay, 10^5^ macrophages/well were incubated with different concentrations of the *C. cajucara* essential oil (1–1000 μg/mL) at 37°C and 5% CO2 for 48 h. Cell viability was assessed after 4 h incubation with resazurin as described by Al-Musayeib *et al.*[12].

### Macrophage infection and NO production

Parasites and/or peritoneal mouse macrophages were pretreated with 250 and 125 μg/ml (MIC and subMIC) of red sacaca essential oil*.* After 20 min, adherent cultured macrophages and free parasites were washed once, resuspended in fresh RPMI culture medium, and then co-cultured at a ratio of 10 promastigotes to 1 macrophage at 37°C for 120 min in a 4% CO_2_ atmosphere. Cells were fixed and Giemsa stained, and the percentage of infected macrophages was determined by counting 600 cells. The association indices were determined by multiplying the percentage of infected macrophages by the mean number of parasites per infected cell. The association index was considered as the number of parasites that successfully infected macrophages. Nitrite levels in culture supernatants of macrophages infected or not with *L. chagasi* were analyzed by Griess reaction as previously described [9]. The absorbance at 550 nm was measured, and the concentration of nitrite was calculated using a linear regression of a standard curve.

### Anti-intracellular amastigote activity

The effects of the essential oil from red sacaca (250 and 125 μg/mL) on intracellular amastigotes were determined after treatment of pre-infected macrophages as previously described with slight modifications [13]. Briefly, mouse peritoneal macrophages were infected with *L. chagasi* promastigotes (logarithmic growth phase) as described above. Next, free promastigotes were removed by extensive washing with PBS and the infected macrophages were then incubated for 24 h to allow complete promastigote differentiation into intracellular amastigotes. Then, MIC and subMIC concentrations of the essential oil were added to the cultures of infected macrophages for 20 min. After treatment, supernatants were collected for the analysis of NO production and coverslips were fixed as previously described above.

All experiments were performed in triplicate. All results are presented as the mean and standard error of the mean (SEM). Normalized data were analyzed by one-way analysis of variance (ANOVA), and differences between groups were assessed using the Student-Newman-Keuls post-test. Results were considered significant at p ≤ 0.05.

## Results and discussion

The crucial role that traditional medicine plays in health care of people living in developing countries is recognized worldwide. For centuries, traditional medicine was the only health care system available for the prevention and treatment of several diseases in different cultures [14]. The research on natural products around the world has found literally thousands of phytocompounds that are biologically active against various a number of illnesses, including infectious diseases. In this study, we described the cytocidal activity of the 7-hydroxycalamenene-rich essential oil from *C. cajucara*, red sacaca, against the etiological agent of Kala-azar, *L. chagasi*.

Most research efforts into the effects of plants on parasite infections have been carried out using aqueous or alcoholic extractions, but purified plant essential oils are also effective in the treatment and prevention of parasitic diseases [15]. Indeed, the essential oils constitute a potential source of bioactive compounds against *Leishmania* species. The linalool-rich essential oil of *C. cajucara*, white sacaca, has been previously described as a potent agent against *L. amazonensis*[9]. The essential oil eliminated 100% of the promastigote and amastigote forms of *L. amazonensis* at a concentration of 15 ng/mL. These results encouraged us to extend the study of the antiprotozoal activity of another *C. cajucara* morphotype, red sacaca, rich in 7-hydroxycalamenene, against *L. chagasi*. The minimum inhibitory concentration (MIC) of *C. cajucara* essential oil and its 7-hydroxycalamenene purified fraction against *L. chagasi* promastigotes was 250 and 15.6 μg/mL, respectively (Table 1). After 70 min of treatment, all parasites were destroyed by the MIC of the essential oil (IC_50 _= 66.7 μg/mL). Several essential oils extracted from different plants have shown antileishmanial activities. A progressive inhibition of parasites was observed when promastigote and amastigote forms of *L. amazonensis* were treated with 100 μg/mL of the eugenol-rich essential oil from *Ocimum gratissimum* (white basil) and its major compound [16]. *Cymbopogon citratus* (lemon grass) showed a marked effect on the viability of *L. infantum* (IC_50_/ 24 h = 25 μg/mL), *L. tropica* (IC_50_/ 24h = 52 μg/mL), and *L. major* (IC_50_/ 48h = 38 μg/mL) promastigotes [17]. *Echinops kebericho* exerted a potent leishmanicidal effect against *L. donovani* and *L. aethiopica* species, with MIC values of 0.0765 μg/mL and 0.0097 μg/mL, respectively [18]. The mechanisms underlying the antileishmanial activity of essential oils are not fully understood. Their lipophilic character, as well as that of their constituents, are possibly involved in the antimicrobial mechanisms by permeating cell membranes and disrupting the structure of the different layers of membrane polysaccharides, fatty acids, and phospholipids, leading to serious cell damage [19].

**Table 1:** ***In vitro *activity of *C. cajucara *(red sacaca) essential oil and its purified 7-hydroxycalamenene-rich fraction against *L. chagasi *promastigotes and toxicity profile in mouse peritoneal macrophages**

**Substance tested**	***L. chagasi***		**Cytotoxicity [μg/mL]**
	**MIC [μg/mL]**	**IC**_**50 **_**[μg/mL]**	
Essential oil	250	66.7	>500
7-hydroxycalamenene purified fraction	15.6	1.37	>500
Amphotericin B	0.625	0.01	14.6

**Figure 2:**
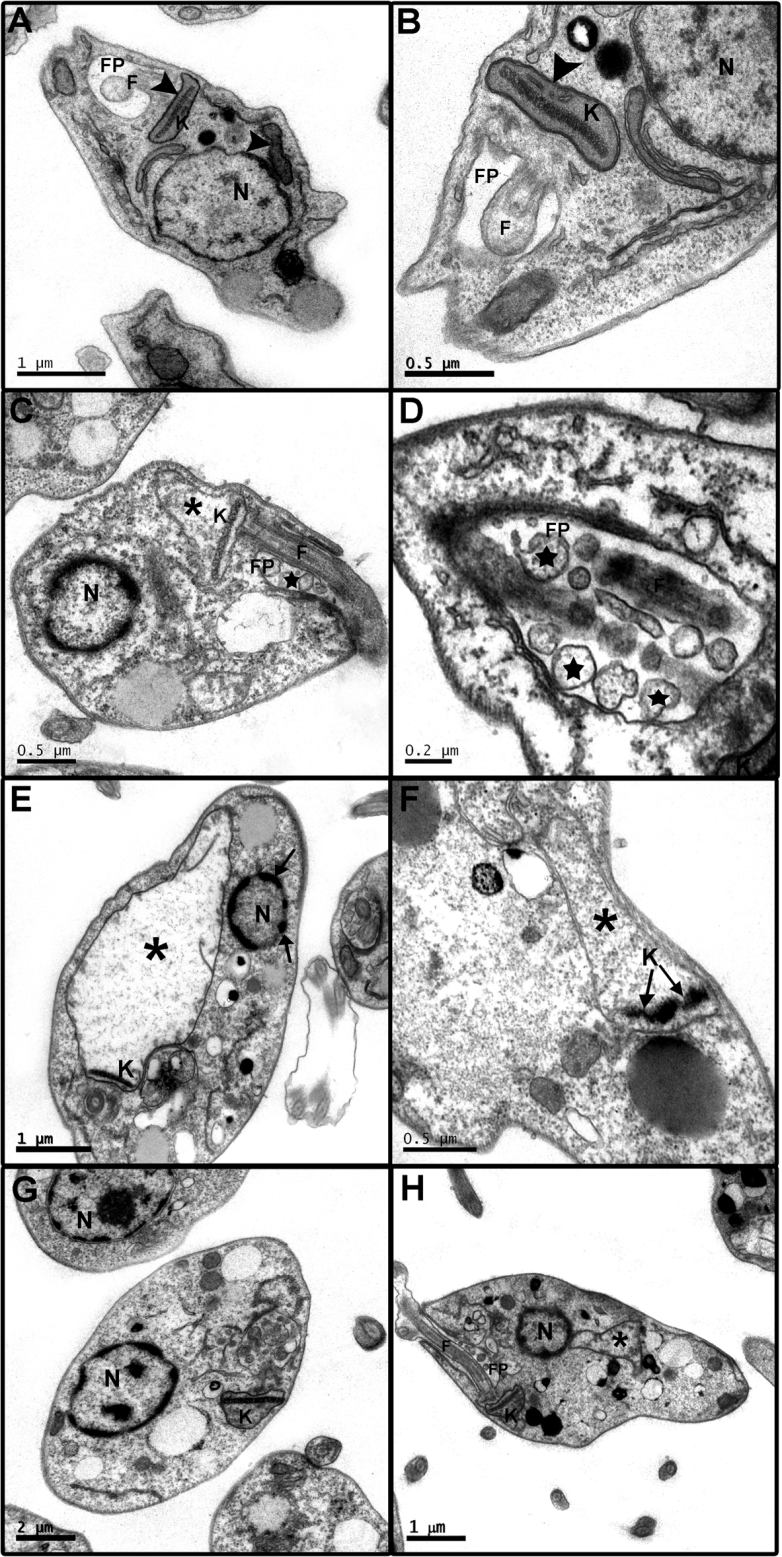
**Ultrastructure alterations induced by the essential oil from *Croton cajucara *in *L. chagasi *promastigotes. (A-B)** Sections of untreated promastigote forms showing the main structures observed under transmission electron microscopy. **(A)** Normal flagellum (F) and flagellar pocket (FP) are observed in the anterior portion of the parasite. The nucleus (N) is round and the mitochondrion (M) is branched and observed along the parasite body, next to the cell surface (head arrows); **(B)** Detail of the mitochondrion containing the kinetoplast (K) with the array of DNA filaments (arrows); **(C-H)** Parasites treated for 5 **(C-D)**, 15 **(E-F)**, and 30 **(G-H)** min with 250 μg/mL of essential oil, showing different degrees of cell damage. **(C)** Parasite presenting dilated mitochondria (*) and condensed nuclear chromatin; **(D)** In the detail, the flagellar pocket showing intense release of vesicles (stars); **(E)** Increased mitochondrial volume and loss of cristae (*) and nuclear chromatin condensation (arrows); **(F)** Increased mitochondrial volume (*) and kDNA fragmentation (arrows) can be noted. **(G-H)** Parasites also exhibit dilated mitochondria (*), condensed nuclear chromatin, flagellar pocket (FP) with intense release of vesicles, and numerous vesicles in the cytoplasm.

Photomicrographs of *L. chagasi* treated with 250 μg/mL of the essential oil from *C. cajucara* are shown in Figure 2 A to H. Depending on the period of treatment, the parasites showed different degrees of cell damage. Within the first 5 min of incubation, increased mitochondrial volume and loss of mitochondrial cristae can be observed (Figure 2C), as well as the presence of vacuoles in the flagellar pocket (Figure 2D). Mitochondrial damage was followed by kDNA fragmentation and condensation of nuclear chromatin (Figures 2E-F), although no changes were observed in the shape of the nucleus. After 30 min of treatment, the cells exhibited complete disorganization of the cytoplasmic organelles (Figure 2G). In addition, dilated mitochondria (*), flagellar pocket with intense release of vesicles, and numerous vesicles in the cytoplasm are noted. Similar mitochondrial swelling and important alterations in the organization of the nuclear and kinetoplast chromatin were observed when *L. amazonensis* was treated with linalool-rich essential oil from *C. cajucara*[9]. Also, the essential oil of *C. citratus* and its major component citral induced changes in the morphology and ultrastructure of *L. amazonensis* promastigotes, such as mitochondrial swelling, the presence of two or more flagella, and exocytic projections in the flagellar pocket [20]. Such types of ultrastructural alterations in *L. amazonensis* have been reported to be associated with the depletion of ergosterol and the alteration of physical properties of the membrane [21]. Indeed, the inhibitory activity of the sesquiterpene nerolidol in the initial steps of the mevalonate pathway, consequently inhibiting isoprenoid biosynthesis (dolichol, ubiquinone, and ergosterol), has been previously demonstrated [22]. Based on the ultrastructural alterations reported here, it is possible that the 7-hydroxycalamenene-rich essential oil from *C. cajucara* exerts similar inhibitory effect on the biosynthesis of isoprenoids. The inhibitory effects of natural products on trypanosomatids enzymes have been reviewed recently [23], highlighting a modern approach to the discovery of new drugs against trypanosomiasis and leishmaniasis.

**Figure 3:**
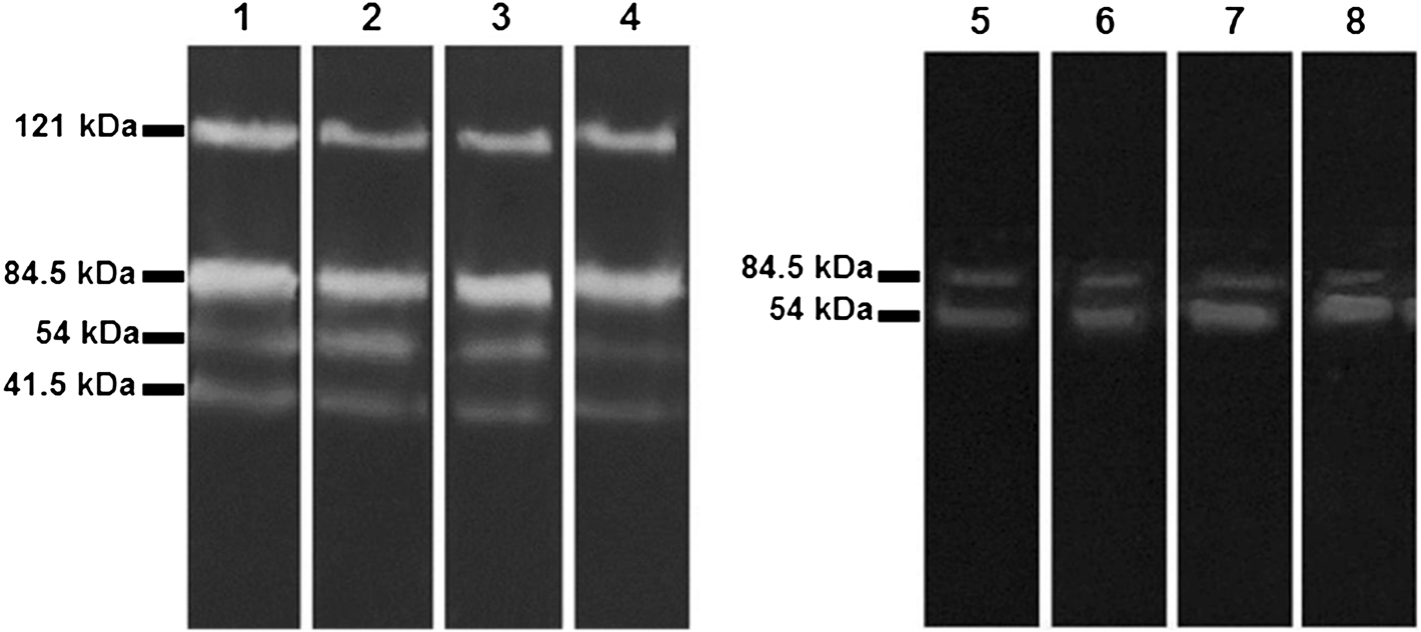
**Peptidase activity of *L. chagasi *promastigotes treated with the essential oil from *C. cajucara*.** Lane 1, untreated parasite lysates (control); Lanes 2–4, lysates of parasites treated for 20 (Lane 2), 40 (Lane 3), and 60 min (Lane 4) with 250 μg/mL of the essential oil. Lane 5, supernatant from untreated parasites (control); Lanes 6–8, supernatants from parasites treated for 20 (Lane 6), 40 (Lane 7), and 60 min (Lane 8) with the essential oil.

As shown in Figure 2D, the presence of vacuoles in the flagellar pocket region of treated promastigotes indicates an intense exocytic activity. This was confirmed by analyzing the presence of peptidases in the supernatants from parasites treated with 250 μg/mL of the essential oil. The analysis of peptidase activity revels that one-hour exposure to the essential oil increased the activity (41%) of only one group of peptidases of 54 kDa (Figure 3). *L. chagasi* promastigote lysates presented a similar 54 kDa protein band, but no significant difference in peptidase activity was noted between treated and untreated cells. The inhibitory effect of E-64 and phenanthroline on *L. chagasi* proteolytic activity confirmed that e 54- and the 41.5-kDa peptidases belong to cysteine peptidases class. The exocytic activity of *L. amazonensis* treated with parthenolide, a sesquiterpene lactone isolated from *Tanacetum parthenium*, has been previously described [24] as an attempt of cell survival. High levels of enzymatic activity were detected in cell lysates but, unlike in our study, the enzymatic activity was not evaluated in the supernatants from parasites.

**Figure 4:**
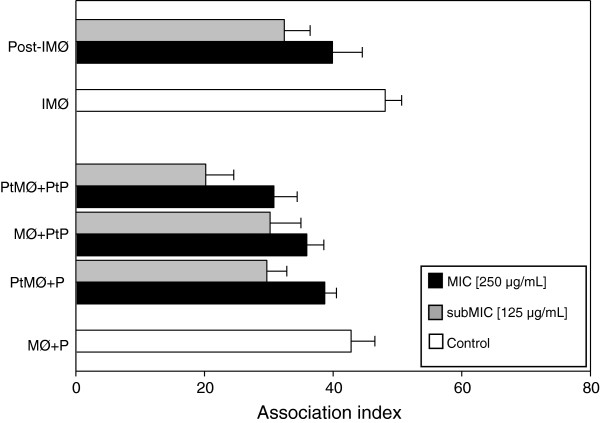
**Effects of 7-hydroxycalamenene-rich essential oil extracted from *C. cajucara *on the interaction of *L. chagasi *with mouse peritoneal macrophages.** Parasites and/or macrophages were treated with MIC (250 μg/mL) or subMIC (125 μg/mL) of red sacaca essential oil 20 min prior to macrophage–parasite interaction. In addition, macrophages previously infected with *L. chagasi* were treated with the essential oil for 20 min and then incubated for 90 min at 37°C in a 5% CO_2_ atmosphere. After 120 min of interaction, association indices were determined by counting 600 cells in triplicate coverslips. Each bar represents the mean ± standard error from at least three independent experiments, each performed in triplicate. (MØ) macrophages; (P) parasites; (PtMØ) pre-treated macrophages; (PtP) pre-treated parasites; (IMØ) infected macrophages; (Post-IMØ) post-treated infected macrophages.

**Figure 5:**
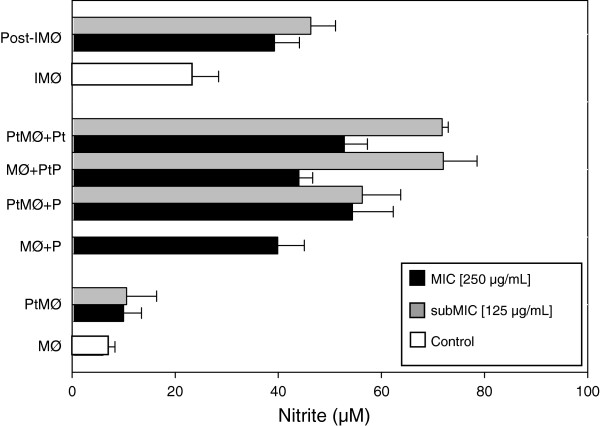
**Effects of 7-hydroxycalamenene-rich essential oil extracted from *C. cajucara *on nitric oxide production by infected mouse peritoneal macrophages.** Parasites and/or macrophages were treated with MIC (250 μg/mL) or subMIC (125 μg/mL) of red sacaca essential oil 20 min prior to macrophage–parasite interaction. In addition, macrophages previously infected with *L. chagasi* were treated with the essential oil for 20 min and then incubated for 120 min at 37°C in a 5% CO_2_ atmosphere. The supernatants from controls (untreated cultures) and *L. chagasi-*infected macrophages were collected, and the nitrite concentration was determined by the Griess reaction. Each bar represents the mean ± standard error from at least three independent experiments, each performed in triplicate. (MØ) macrophages; (P) parasites; (PtMØ) pre-treated macrophages; (PtP) pre-treated parasites; (IMØ) infected macrophages; (Post-IMØ) post-treated infected macrophages.

Some essential oils from plants have immunomodulatory effects that are useful in the treatment of infectious diseases, particularly when oils cause no adverse effects to the host. All concentrations of the essential oil from red sacaca used in this study did not show toxicity to mouse peritoneal macrophages. Figure 4 shows the effects of the essential oil on *L. chagasi*–macrophage interaction. In all systems tested, it not only reduced the association index but it also increased NO production by infected macrophages (Figure 5). In the present work, we observed that pretreatment of macrophages with 250 and 125 μg/mL of essential oil reduced the number of adherent and internalized parasites (Association Indices) in 30.0 and 9.6%, respectively. In these conditions, NO production was 41.1 and 36.3% higher, respectively. Pre-treatment of parasites with the same concentrations of the essential oil decreased the association indices by 29.4 and 16.1% and increased NO production by 80.4 and 10%, respectively. Pre-treatment of both promastigotes and macrophages reduced the association indices by 52.8 and 28%, and increased NO production by 80 and 32.5%, respectively. When macrophages were pre-infected with *L. chagasi* for 24 hours and then treated with 250 μg/mL of the essential oil, the association index was 32.7% lower and NO production was 100% higher than control. Similar effects on *L. amazonensis*–macrophage interaction have been previously demonstrated for white sacaca [9], *basil*[16], and *lemongrass* essential oils [20]. Furthermore, the addition of *Aloe vera* exudate to *L. donovani*-infected macrophages significantly increased NO production compared to untreated macrophages [25]. On the other hand, despite its potent leishmanicidal activity, the essential oil from *Chenopodium ambrosioides* has been shown to inhibit the phagocytic activity of *L. donovani*-infected macrophages by only 17.6% [26].

Most of the conventional antileishmanial drugs exhibit strong *in vitro* activity against the parasites but they are also highly toxic for mammalian host cells. By contrast, several studies have demonstrated that crude essential oils and their major compounds present low or no toxicity to host cells at the effective concentrations [27-30]. Both the essential oil and its 7-hydroxycalamenene-rich purified fraction presented no toxicity for peritoneal mouse macrophages at the concentrations used in this study.

## Conclusion

Our results further support the antileishmanial activity of 7-hydroxycalamenene-rich essential oil of *C. cajucara* (red sacaca) leaf extracts. Its toxicity against *L. chagasi*, with no effect on mammalian cells, indicates that this essential oil is a promising source of antileishmanial agents. Further studies, including *in vivo* bioassays, are needed to validate the *in vitro* results and to ascertain the safety of the essential oil and the 7-hydroxycalamenene purified fraction.

## Competing interests

The authors declare that they have no competing interests.

## Authors’ contributions

IAR and MMBA: conceived and designed the work. MMBA carried out the chromatography analysis and isolation of 7-hydroxycalamenene. IAR performed the antileishmanial experiments, analyzed the data and wrote the manuscript; FCMC: provided the plant material and carried out the essential oil extraction; HRB: carried out the GC/MS analysis and provided part of the reagents/analytical tools; SCR: was responsible for the transmission electron microscopy and data analysis; DSA and CSA: assisted in the design of the work and provided part of the reagents; MSSR: contributed in the analysis and interpretation of antileishmanial data; ABV: is the principal investigator, supervised IAR and contributed in the analysis and interpretation of data. All authors gave their approval for the final version of the manuscript to be published.

## Pre-publication history

The pre-publication history for this paper can be accessed here:

http://www.biomedcentral.com/1472-6882/13/249/prepub
